# Dry Eye Disease Patients with Xerostomia Report Higher Symptom Load and Have Poorer Meibum Expressibility

**DOI:** 10.1371/journal.pone.0155214

**Published:** 2016-05-05

**Authors:** Ida G. Fostad, Jon R. Eidet, Tor P. Utheim, Sten Ræder, Neil S. Lagali, Edvard B. Messelt, Darlene A. Dartt

**Affiliations:** 1 Department of Oral Biology, University of Oslo, Oslo, Norway; 2 The Norwegian Dry Eye Clinic, Oslo, Norway; 3 Department of Ophthalmology, Oslo University Hospital, Oslo, Norway; 4 Unit of Regenerative Medicine, Department of Medical Biochemistry, Oslo University Hospital, Oslo, Norway; 5 Department of Ophthalmology, Vestre Viken Hospital Trust, Drammen, Norway; 6 Faculty of Health Sciences, Buskerud and Vestfold University College, Kongsberg, Norway; 7 Department of Ophthalmology, Institute for Clinical and Experimental Medicine, Linköping University, Linköping, Sweden; 8 Schepens Eye Research Institute, Massachusetts Eye and Ear/Harvard Medical School, Boston, MA, United States of America; Save Sight Institute, AUSTRALIA

## Abstract

The purpose of the study was to investigate if xerostomia (dry mouth) is associated with symptoms and signs of dry eye disease (DED). At the Norwegian Dry Eye Clinic, patients with symptomatic DED with different etiologies were consecutively included in the study. The patients underwent a comprehensive ophthalmological work-up and completed self-questionnaires on symptoms of ocular dryness (Ocular Surface Disease Index [OSDI] and McMonnies Dry Eye Questionnaire) and the Sjögren’s syndrome (SS) questionnaire (SSQ). Three hundred and eighteen patients (52% women and 48% men) with DED were included. Patient demographics were: 0 to 19 years (1%), 20 to 39 (25%), 40 to 59 (34%), 60 to 79 (35%) and 80 to 99 (5%). Xerostomia, defined as “daily symptoms of dry mouth the last three months” (as presented in SSQ) was reported by 23% of the patients. Female sex was more common among patients with xerostomia (81%) than among non-xerostomia patients (44%; *P*<0.001). Patients with xerostomia (60 ± 15 years) were older than those without xerostomia (51 ± 17; *P*<0.001). The use of prescription drugs was more prevalent among xerostomia patients (65%) than among non-xerostomia patients (35%; *P*<0.021; adjusted for age and sex). Patients with xerostomia had a higher OSDI score (19.0 ± 10.0) than those without xerostomia (12.9 ± 8.0; *P*<0.001). Moreover, xerostomia patients had more pathological meibum expressibility (0.9 ± 0.7) than those without xerostomia (0.7 ± 0.8; *P* = 0.046). Comparisons of OSDI and ocular signs were performed after controlling for the effects of sex, age and the number of systemic prescription drugs used. In conclusion, xerostomia patients demonstrated a higher DED symptom load and had poorer meibum expressibility than non-xerostomia patients.

## Introduction

Ocular dryness and xerostomia are common symptoms in the population for which many patients seek medical help for their condition [1]. According to the dry eye workshop (2007) “Dry eye is a multifactorial disease of the tears and ocular surface that results in symptoms of discomfort, visual disturbance and tear film instability with potential damage to the ocular surface. It is accompanied by increased osmolarity of the tear film and inflammation of the ocular surface” [2]. Systemic diseases (Sjögren’s syndrome [SS] and diabetes), as well as local (contact lens wear) and environmental (low humidity) factors, can cause dry eye disease (DED) [2]. The prevalence of DED is estimated to 5–30% in the population above 50 years of age [2]. Dependent on etiology, DED is divided in two major categories; an aqueous deficient and an evaporative type. These two categories do not mutually exclude each other as they can act in combination.

Xerostomia is defined as a subjective feeling of dry mouth. Patients with this condition may report substantial discomfort and reduced quality of life [3]. Some complain of difficulties with swallowing, speaking, burning mouth and wearing dentures [4]. The term salivary dysfunction is herein used to indicate patients with xerostomia and/or patients whose saliva flow rate is measurably reduced [5]. Since saliva has many important properties in the oral cavity, dysfunction may cause devastating oral effects, such as caries, erosions and infections [6].

Symptoms and clinical findings do not always correspond, as patients with xerostomia do not always have reduced salivary output [7]. The same lack of relation is also found among patients with ocular dryness, whose clinical signs of DED are not always detected [8–10]. The presence of xerostomia and ocular dryness has traditionally been included among the diagnostic criteria for SS [11, 12]. However, a new revision of the diagnostic criteria for SS is suggested, since ocular dryness and xerostomia are common conditions and individually have low specificity for diagnosing SS [1, 13].

Several factors and conditions can induce xerostomia. Use of prescription drugs is one of the most common causes [14]. In addition to SS, a wide variety of autoimmune and endocrine disorders are associated with xerostomia [15]. These diseases include diabetes, rheumatoid arthritis, systemic lupus erythematosus and thyroid disease, even when they are not co-occurring with SS [16]. All of these conditions are also associated with DED [2, 17–19].

Schein *et al*. investigated the prevalence of xerostomia and/or dry eyes in a population based study that included 2481 individuals between 65 and 85 years [20]. Akpek and associates evaluated the number of patients with an underlying systemic disease in a cohort of DED patients, but they did not report the prevalence of xerostomia [21]. Neither the study by Schein *et al*. [20] nor the one by Akpek and associates [21] investigated if xerostomia is associated with the degree of severity of symptoms and signs of DED. If such an association can be found, xerostomia might be a more useful anamnestic marker when examining DED patients than previously considered.

In this study, we assessed the prevalence of xerostomia in 318 patients with DED from 15 to 91 years of age. We compared patients with and without xerostomia to explore whether they differed in the degree of symptoms and signs of DED.

## Materials and Methods

### Patients

The study was conducted in accordance with the Declaration of Helsinki. Three hundred and eighteen patients with dry eyes, diagnosed by an ophthalmologist at the Norwegian Dry Eye Clinic between August 2012 and October 2013, that did not have missing data on any of the study parameters, were included. The patients underwent a comprehensive ophthalmological work-up and completed self-report questionnaires on symptoms of ocular dryness (Ocular Surface Disease Index [OSDI] and McMonnies Dry Eye Questionnaire) and the SS questionnaire. The questionnaires were distributed and collected at the Norwegian Dry Eye Clinic by the examining ophthalmologist, who also co-authored. The data from the questionnaires were then made anonymous by the same ophthalmologist, and included in the Norwegian Dry Eye Clinic databank. All examinations were carried out at the same clinic. The use of the data for the study from the Norwegian Dry Eye Clinic has been reviewed by The Regional Committee for Medical & Health Research Ethics, Section C, South East Norway (REC). REC found the research project “*Evaluation of data from the Norwegian Dry Eye Clinic*” to be outside the remit of the Act on Medical and Health Research (2008) and therefore can be implemented without its approval. A REC letter of exemption has been provided.

### Ophthalmological Work-Up

The same ophthalmologist examined all patients during normal working hours between 9 AM and 4 PM. The ophthalmological examination included assessment of ocular staining (lissamine green and fluorescein) [22], tear film break-up time (TFBUT) [23, 24], Schirmer I [18], meibum expressibility [25], meibum quality [25], ocular protection index (OPI) [24], and dry eye severity level (DESL) (Table 1) [18]. The mean scores from both eyes of each subject were used for analyses. Measurements were performed on patients irrespective of degree of dry eye severity.

**Table 1:** Ophthalmological Work-up.

Parameter	Scoring Method	Pathological Score
**Dry eye severity level [2]**	Four-level composite score based on ocular discomfort, visual disturbance, conjunctival injection, conjunctival/corneal staining, other signs of corneal/tear pathology, signs of lid/meibomian gland pathology, TFBUT and Schirmer score.	>0
**Tear film break-up time (TFBUT) [23, 24]**	The interval in seconds between the last complete blink and the first appearance of a dry spot, or disruption in the tear film following instillation of fluorescein.	≤10
**Ocular protection index [24]**	TFBUT divided by the interblink interval (mean time in seconds between two complete blinks).	<1
**Schirmer I [18]**	Paper test strips are inserted in the lower lateral third of the conjunctival sac and the eyes are closed for 5 minutes. The wetting of the paper strip is then measured in millimeters.	≤10
**Staining [22]**	Following fluorescein instillation the staining scores of the exposed cornea and interpalpebral conjunctiva are summarized using the Oxford grading scheme (range: 0–15).	>0
**Meibum expressibility [25]**	Five glands in the lower lid are evaluated according to the number of expressible glands: 0, all glands; 1, three to four glands; 2, one to two glands; and 3, no glands (score range: 0 to 3).	>0
**Meibum quality [25]**	Eight glands from the central third of the lower lid are evaluated on a scale of 0 to 3 for each gland: 0, clear; 1, cloudy; 2, cloudy with debris (granular); and 3, thick, toothpaste-like (total score range: 0–24).	>0

### Self-Report Questionnaires

The OSDI questionnaire included 12 items regarding symptoms of ocular dryness (Table 2) [26]. The SS questionnaire incorporated six items on ocular and oral symptoms by the revised criteria from the American- European consensus group [12]. In the current study, patients answering “yes” to the question “have you had daily sensation of dry mouth the last three months” in the SS questionnaire were defined as suffering from xerostomia. For each patient the number of different systemic prescription drug categories used, as defined by the McMonnies Dry Eye Questionnaire, was summarized (Table 3).

**Table 2:** Ocular Surface Disease Index.

*Have you experienced any of the following during the last week*?
1. Eyes that are sensitive to light?
2. Eyes that feel gritty?
3. Painful or sore eyes?
4. Blurred vision?
5. Poor vision?
*Have problems with your eyes limited you in performing any of the following during the last week*?
6. Reading?
7. Driving at night?
8. Working with computer screens?
9. Watching TV?
*Have your eyes felt uncomfortable in any of the following situations during the last week*?
10. Windy conditions?
11. Places or areas with low humidity (very dry)?
12. Areas that are air-conditioned?

**Table 3:** Prescription drug categories used in xerostomia and non-xerostomia patients.

Prescription drug categories^1^	Xerostomia^2^ (n = 72)	Non-xerostomia (n = 246)	XerostomiaOR (95% CI)	*P*-value^3^
	Number of patients (%)	Number of patients (%)		
Antihistamines	22 (31)	27 (11)	***2*.*8 (1*.*4–5*.*6)***	***0*.*004***
Diuretics	7 (10)	5 (2)	***4*.*0 (1*.*1–14*.*2)***	***0*.*033***
Antidepressants	12 (17)	13 (5)	2.1 (0.8–5.2)	0.109
Anxiolytics	12 (17)	26 (11)	0.8 (0.3–1.7)	0.492
Medications against urge incontinence	3 (4)	3 (1)	1.7 (0.3–9.6)	0.529
Oral contraceptives	1 (2)	9 (8)	0.5 (0.1–4.6)	0.550^4^
Antihypertensives	8 (11)	11 (5)	1.3 (0.5–3.8)	0.615
Antitussives	8 (11)	20 (8)	1.3 (0.5–3.2)	0.625
Medications against dyspepsia and gastro-esophageal reflux	5 (7)	10 (4)	1.2 (0.4–4.1)	0.771
Anti-Parkinsonian medications	1 (1)	1 (0)	1.2 (0.1–20.4)	0.903
Hormone replacement therapy	7 (12)	9 (8)	1.1 (0.4–3.2)	0.908^4^
Medications against chronic obstructive pulmonary disease	1 (1)	1 (0)	0.9 (0.1–14.8)	0.922
Medications against benign prostatic hyperplasia	0 (0)	4 (3)	0 (0)	0.999^5^
Medications against acne	0 (0)	1 (0)	0 (0)	1.0
Antipsychotics	0 (0)	0 (0)	-	-

OR = odds ratio; CI = confidence interval

^1^As defined by the McMonnies Dry Eye Questionnaire.

^2^Daily dry mouth for the last three months

^3^Logistic regression analysis adjusted for age and sex, unless otherwise stated.

^4^Analysed in female patients (n = 166) and adjusted for age.

^5^Analysed in male patients (n = 152) and adjusted for age.

### Statistics

Data are presented as mean ± standard deviation (SD). Chi-square test was performed when comparing differences in sex, prescription drug use and meibum expressibility between patients with and without xerostomia, whereas Student’s t-test was used to compare the age of patients with and without xerostomia. The use of various systemic prescription drugs was compared between patients with and without xerostomia using logistic regression analysis with adjustment for age and sex. To assess the differences in OSDI and ocular signs between the patients with and without xerostomia, multivariate analysis of covariance (MANCOVA) was performed, adjusting for age, sex and the number of systemic prescription drugs used. A significance level of *P*<0.05 was used throughout the study (SPSS ver. 21.0).

## Results

**Fig 1:**
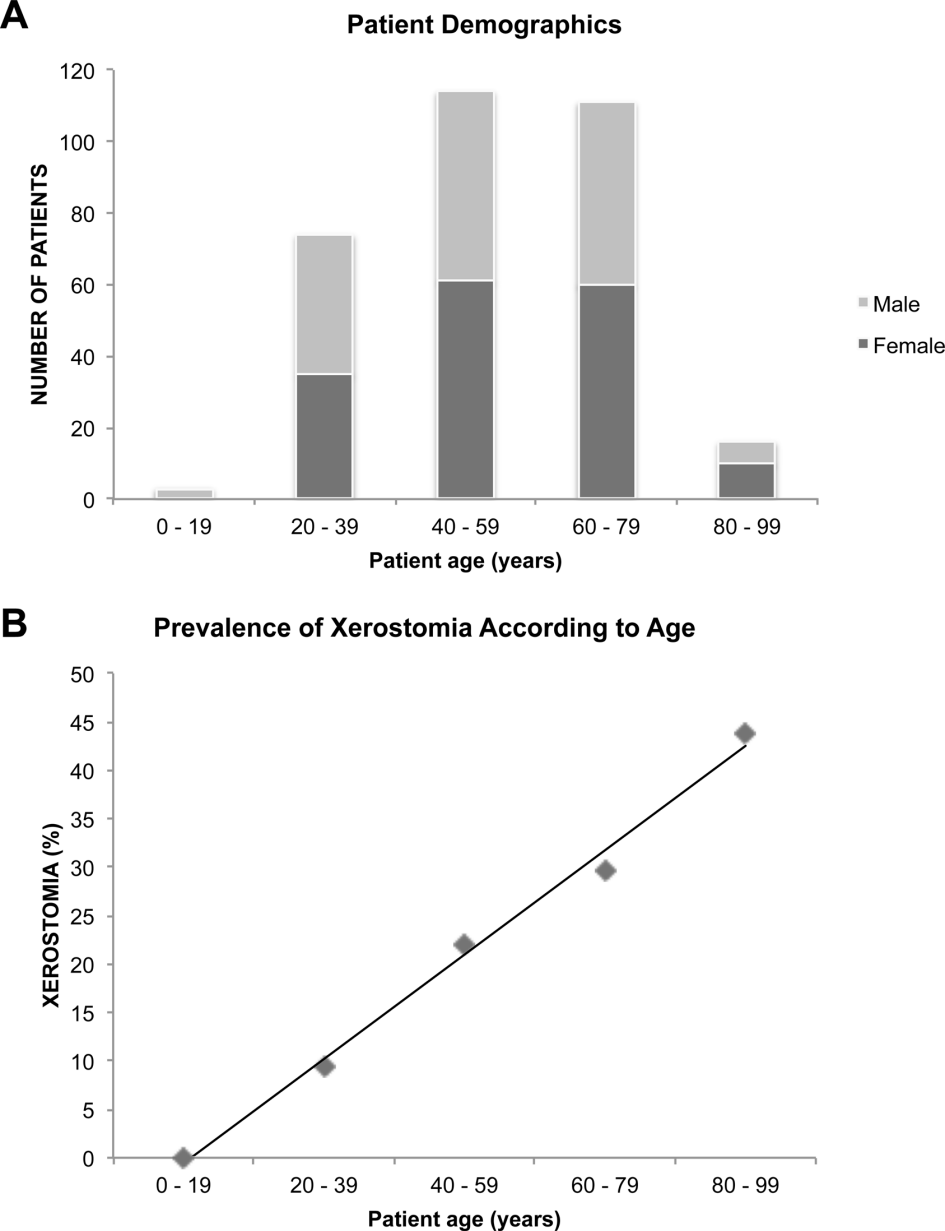
Patient demographics. (**A**) Bar graph showing the age and sex distribution among 318 patients with dry eye disease consecutively included in the study. (**B**) Scatterplot showing the prevalence of xerostomia according to age in 318 dry eye disease patients.

The majority of the patients (71%) were between 40 and 80 years of age, with a similar sex distribution across most age groups (Fig 1A). Xerostomia was confirmed by 72 of the 318 patients (23%). One hundred and sixty-six (52%) of the 318 patients were females and 152 (48%) were males. Seven of the 318 patients (2%) reported a previous SS diagnosis.

Compared to those without xerostomia, patients reporting xerostomia were overrepresented by women (44% and 81% women, respectively; *P*<0.001). Sixty-five percent of patients with xerostomia, in contrast to only 35% of patients without xerostomia, used at least one prescription drug (*P*<0.021; adjusted for age and sex). The use of antihistamines and diuretics were more prevalent among xerostomia patients than among non-xerostomia patients (*P*<0.05; adjusted for age and sex) (Table 3). Patients with xerostomia (60 ± 15 years) were older than those without xerostomia (51 ± 17; *P*<0.001) (Fig 1B).

Patients with xerostomia were compared with those without xerostomia on the presence of symptoms and signs of DED following adjustment for age, sex and the number of systemic prescription drugs used (Table 4). After applying a threshold to separate those with normal meibum expressibility (all glands expressible, which equals a score of 0) from those with pathological meibum expressibility (not all glands expressible, which equals a score >0) the Chi-square test was also used to investigate the association between xerostomia and meibum expressibility. Among the 72 patients with xerostomia 72% (52 patients) had pathological meibum expressibility, whereas among the 246 patients without xerostomia, only 53% (130 patients) had pathological meibum expressibility (*P* = 0.003). Thus, these results show that the xerostomia patients have a more severe meibum expressibility score and a lower prevalence of normal meibum expressibility.

**Table 4:** Severity of Ocular Signs in Patients with/without Xerostomia.

Parameter	Xerostomia^1^	Non-xerostomia	F	P-value*
Ocular surface disease index (OSDI)	19.0 ± 10.0	12.9 ± 8.0	***20*.*2***	***<0*.*001***
Dry eye severity level (DESL)	2.1 ± 0.5	2.0 ± 0.5	1.8	0.183
Tear film break up time (TFBUT)	5.7 ± 3.6	6.5 ± 4.2	0.1	0.760
Ocular protection index (OPI)	2.2 ± 1.8	2.5 ± 2.1	0.2	0.688
Schirmer I	13.4 ± 9.2	15.0 ± 9.0	2.2	0.141
Staining	1.8 ± 2.0	1.5 ± 1.9	0.9	0.356
Meibum expressibility	0.9 ± 0.7	0.7 ± 0.8	***4*.*0***	***0*.*046***
Meibum quality	5.4 ± 3.3	6.4 ± 4.8	0	0.867

^1^ Daily dry mouth for the last three months

* Multivariate analyses of covariance with adjustment for age, sex and the number of systemic prescription drugs used.

## Discussion

In the current study, we found that 23% of the DED patients reported xerostomia, and that xerostomia patients, compared to those without xerostomia, reported a higher symptom load and had poorer meibum expressibility. Hence, these results could indicate that, in patients with DED, reporting xerostomia is a risk factor for more pronounced symptoms of dry eyes.

The prevalence of xerostomia varies between the population studied and depends on how xerostomia is defined in each study. This issue is elucidated by Hopcraft and Tan [27]. They found that the prevalence of xerostomia in population based studies varied between 10 to 46% [27]. A study by Schein *et al*. showed that ocular dryness *or* xerostomia were present often or all the time in 27% in a population-based study involving 2481 individuals between the ages of 65 to 85 years [20]. Only 4.4% reported to have concomitant ocular dryness *and* xerostomia [20]. In our study, 23% (33% of patients between 65 and 85 years) of the dry eye patients reported xerostomia. The Schein study [20], however, used a different definition of ‘xerostomia’ compared to the current study.

Schein *et al*. used a symptom-based definition of dry eyes. Their six-item dry eye questionnaire [28] included questions regarding the sensation of dry, gritty, red or burning eyes, as well as lash crusting and the experience of eyes getting stuck shut in the morning. Degree of symptomatic dry eyes in the current study was scored using the OSDI, which includes 12 validated questions for measuring the severity of DED (Table 2) [29]. Whereas the questionnaire used by Schein *et al*. and the OSDI both include questions regarding symptoms directly associated with dry eyes, the latter also includes items on vision-related function. In addition to symptoms, the current study also included clinical tests to diagnose DED (Table 1). Patients having a DESL of at least 1, as determined by the examining ophthalmologist, were defined as having DED. DESL is a four-level composite score based on symptoms and signs of dry eye (Table 1).

In the current study, clinical tests were performed irrespective of the degree of dry eyes or any other known patient characteristics. Hence, missing data were assumed missing completely at random. Patients that did not have complete data sets were therefore excluded from the study by listwise deletion. Nevertheless, as patients with incomplete data sets were excluded a potential selection bias cannot be definitely ruled out.

Patients with xerostomia reported a significantly higher OSDI score than patients without xerostomia after adjusting for age, sex, and use of systemic prescription drugs. Interestingly, Alves *et al*. found that OSDI scores were higher in patients with SS, diabetes mellitus and thyroid disorder than control subjects [30]. All of these conditions are also associated with xerostomia [31, 32]. In contrast to our finding that xerostomia is related to the OSDI score, which is a subjective measure of ocular dryness, the correlation between hyposalivation and xerostomia has been reported to be weak [33]. Since we have controlled for several confounders in this study the association between xerostomia and OSDI could, therefore, be attributed partly to higher symptom awareness in some patients or an undiagnosed systemic disease aggravating both xerostomia and DED.

The female/male-ratio was approximately 1:1 in our study. This is somewhat unexpected since DED predominates among women [18]. In addition to ocular dryness, women more often report having xerostomia [27], which was also the case in the current study. Hormonal alterations in the levels of androgen and estrogen are suggested to be related to ocular dryness and xerostomia due to the worsening of these symptoms in postmenopausal women [34, 35].

Increasing age and the use of prescription drugs were associated with xerostomia in the present study, which is also in line with previous reports [27].

The increased prevalence of prescription drug use among patients with xerostomia compared to those without xerostomia was independent of age and sex. Aging of the salivary gland coincides with a reduction in the amount of acini and an increase in fat infiltration and fibrosis [36]. Although increasing age may be linked with xerostomia, the reserve capacity in the salivary glands seems to compensate for these morphological alterations and does not reduce salivation significantly [37–39]. The association between age and xerostomia has often been related to the use of prescription drugs [40]. More than 500 prescription drugs, including anticholinergic, antidepressants and antihistamines, are reported to cause xerostomia [7]. Dosage and duration of prescription drugs used are also important factors [41, 42]. Use of prescription drugs is also related to DED symptoms. We previously reported that use of anxiolytics and antipsycotics demonstrated the strongest correlation with symptomatic dry eyes in DED patients, as assessed by the McMonnie’s Dry Eye Questionnaire and OSDI [43]. In contrast, antihistamines were the most prevalent systemic prescription drug used in xerostomia patients in our study. This is consistent with previous reports that have showed induction of xerostomia by antihistamines [44].

In the current study, among the ocular signs, only meibum expressibility was significantly worse in the xerostomia patients. Meibum quality, however, was not associated with xerostomia. Poor meibum expressibility is one of the hallmarks of evaporative DED caused by meibomian gland dysfunction (MGD), which is the most common form of DED [17]. The sebaceous meibomian glands normally supply lipids and proteins to the outer layer of the tear film. The outer lipid layer decreases the evaporation of the water content, hence is important for tear film stability [18, 45]. The main cause of MGD is ductal orifice obstruction due to hyperkeratinization [25]. The prevalence of MGD increases with age and is influenced by sex [25]. Various endogenous and/or exogenous factors can cause gland obstruction and/or alteration in meibomian gland secretion (quantity and/or quality). Endogenous factors include dysfunction of androgens and estrogens, which regulate the meibomian glands [25]. Exogenous factors include topical eye medications and contact lens use [25]. Since meibum expressibility, but not meibum quality, was affected in patients with xerostomia in our study, patients with xerostomia may exhibit a low delivery, rather than a high delivery, type of MGD [25]. The subtype of MGD dysfunction in patients with xerostomia could be investigated further by meibography and *in vivo* laser confocal microscopy to detect possible gland atrophy and dropout, periglandular inflammatory cell infiltrates and periglandular fibrosis, of which the latter two are typically seen in obstructive MGD [46, 47].

## Conclusion

In the current study DED patients with xerostomia demonstrated a higher symptom load, as demonstrated by the OSDI questionnaire, and more pathological meibum expressibility. Thus, xerostomia in DED patients may be used as an anamnestic indicator of MGD, which could warrant more extensive analyses of meibomian gland function, in addition to considering evaluation for any underlying systemic disease. Future studies using meibography and *in vivo* laser confocal microscopy could shed light on defining the subtype of MGD seen in patients with xerostomia. Xerostomia is an important factor requiring consideration in the assessment of DED.

## Supporting Information

S1 Table Patient Demographics with Symptoms and Signs. (ZIP)

## References

[1] Sankar V, Noll JL, Brennan MT. Diagnosis of Sjogren's syndrome: American-European and the American College of Rheumatology classification criteria. Oral and maxillofacial surgery clinics of North America. 2014;26(1):13–22. 10.1016/j.coms.2013.09.001 24287190

[2] The definition and classification of dry eye disease: report of the Definition and Classification Subcommittee of the International Dry Eye WorkShop (2007). The ocular surface. 2007;5(2):75–92. 17508116 10.1016/s1542-0124(12)70081-2

[3] Cassolato SF, Turnbull RS. Xerostomia: clinical aspects and treatment. Gerodontology. 2003;20(2):64–77. 14697016 10.1111/j.1741-2358.2003.00064.x

[4] Narhi TO, Meurman JH, Ainamo A. Xerostomia and hyposalivation: causes, consequences and treatment in the elderly. Drugs & aging. 1999;15(2):103–16. 10495070 10.2165/00002512-199915020-00004

[5] Davies A. Salivary gland dysfunction In: Davies A, Finlay I, editors. Oral care in advanced disease. Oxford: Oxford University Press; 2005 p. 97–113.

[6] Nederfors T, Isaksson R, Mornstad H, Dahlof C. Prevalence of perceived symptoms of dry mouth in an adult Swedish population—relation to age, sex and pharmacotherapy. Community dentistry and oral epidemiology. 1997;25(3):211–6. 9192149 10.1111/j.1600-0528.1997.tb00928.x

[7] Fox PC. Acquired salivary dysfunction. Drugs and radiation. Annals of the New York Academy of Sciences. 1998;842:132–7. 9599303 10.1111/j.1749-6632.1998.tb09641.x

[8] Sullivan BD, Crews LA, Messmer EM, Foulks GN, Nichols KK, Baenninger P, et al Correlations between commonly used objective signs and symptoms for the diagnosis of dry eye disease: clinical implications. Acta ophthalmologica. 2014;92(2):161–6. 10.1111/aos.12012 23279964

[9] Schmidl D, Witkowska KJ, Kaya S, Baar C, Faatz H, Nepp J, et al The association between subjective and objective parameters for the assessment of dry eye syndrome. Investigative ophthalmology & visual science. 2015. 10.1167/iovs.14-15814 25650419

[10] Bartlett JD, Keith MS, Sudharshan L, Snedecor SJ. Associations between signs and symptoms of dry eye disease: a systematic review. Clin Ophthalmol. 2015;9:1719–30. 10.2147/OPTH.S89700 26396495 PMC4577273

[11] Delli K, Spijkervet FK, Kroese FG, Bootsma H, Vissink A. Xerostomia. Monographs in oral science. 2014;24:109–25. 10.1159/000358792 24862599

[12] Vitali C, Bombardieri S, Jonsson R, Moutsopoulos HM, Alexander EL, Carsons SE, et al Classification criteria for Sjogren's syndrome: a revised version of the European criteria proposed by the American-European Consensus Group. Annals of the rheumatic diseases. 2002;61(6):554–8. 12006334 10.1136/ard.61.6.554 PMC1754137

[13] Shiboski SC, Shiboski CH, Criswell L, Baer A, Challacombe S, Lanfranchi H, et al American College of Rheumatology classification criteria for Sjogren's syndrome: a data-driven, expert consensus approach in the Sjogren's International Collaborative Clinical Alliance cohort. Arthritis care & research. 2012;64(4):475–87. 22563590 10.1002/acr.21591 PMC3349440

[14] Han P, Suarez-Durall P, Mulligan R. Dry mouth: A critical topic for older adult patients. Journal of prosthodontic research. 2014. 10.1016/j.jpor.2014.11.001 25498205

[15] Pedersen AML. Diseases Causing Oral Dryness In: Carpenter G, editor. Dry Mouth. London, UK: Springer; 2015.

[16] Aliko A, Wolff A, Dawes C, Aframian D, Proctor G, Ekstrom J, et al World Workshop on Oral Medicine VI: clinical implications of medication-induced salivary gland dysfunction. Oral surgery, oral medicine, oral pathology and oral radiology. 2015. 10.1016/j.oooo.2014.10.027 25861957

[17] Bron AJ, Tomlinson A, Foulks GN, Pepose JS, Baudouin C, Geerling G, et al Rethinking Dry Eye Disease: A Perspective on Clinical Implications. The ocular surface. 2014;12(2S):S1–S31. 24725379 10.1016/j.jtos.2014.02.002

[18] The Definition and Classification of Dry Eye Disease: Report of the Definition and Classification Subcommittee of the International Dry Eye Workshop (2007). The Ocular Surface. 2007;5(2):75–92. 17508116 10.1016/s1542-0124(12)70081-2

[19] Resch MD, Marsovszky L, Németh J, Bocskai M, Kovács L, Balog A. Dry Eye and Corneal Langerhans Cells in Systemic Lupus Erythematosus. Journal of Ophthalmology. 2015;2015:8. 10.1155/2015/543835 PMC4393942 25893112

[20] Schein OD, Hochberg MC, Munoz B, Tielsch JM, Bandeen-Roche K, Provost T, et al Dry eye and dry mouth in the elderly: a population-based assessment. Archives of internal medicine. 1999;159(12):1359–63. 10386512 10.1001/archinte.159.12.1359

[21] Akpek EK, Klimava A, Thorne JE, Martin D, Lekhanont K, Ostrovsky A. Evaluation of patients with dry eye for presence of underlying Sjogren syndrome. Cornea. 2009;28(5):493–7. 10.1097/ICO.0b013e31818d3846 19421051 PMC2693267

[22] Bron AJ, Evans VE, Smith JA. Grading of corneal and conjunctival staining in the context of other dry eye tests. Cornea. 2003;22(7):640–50. 14508260 10.1097/00003226-200310000-00008

[23] Bron AJ. Diagnosis of dry eye. Survey of ophthalmology. 2001;45 Suppl 2:S221–6. 11587146 10.1016/s0039-6257(00)00201-0

[24] Ousler GW 3rd, Hagberg KW, Schindelar M, Welch D, Abelson MB. The Ocular Protection Index. Cornea. 2008;27(5):509–13. 10.1097/ICO.0b013e31816583f6 18520496

[25] Nichols KK, Foulks GN, Bron AJ, Glasgow BJ, Dogru M, Tsubota K, et al The international workshop on meibomian gland dysfunction: executive summary. Investigative ophthalmology & visual science. 2011;52(4):1922–9. 21450913 10.1167/iovs.10-6997a PMC3072157

[26] Chan C. Practical Office-Based Screening and Diagnostics. In: Chan C, editor. Dry Eye—A Practical Approach. Essentials in Ophthalmology2015. p. 31–44.

[27] Hopcraft MS, Tan C. Xerostomia: an update for clinicians. Australian dental journal. 2010;55(3):238–44; quiz 353. 10.1111/j.1834-7819.2010.01229.x 20887509

[28] Schein OD, Tielsch JM, Munoz B, Bandeen-Roche K, West S. Relation between signs and symptoms of dry eye in the elderly. A population-based perspective. Ophthalmology. 1997;104(9):1395–401. 9307632 10.1016/s0161-6420(97)30125-0

[29] Schiffman RM, Christianson MD, Jacobsen G, Hirsch JD, Reis BL. Reliability and validity of the Ocular Surface Disease Index. Archives of ophthalmology. 2000;118(5):615–21. 10815152 10.1001/archopht.118.5.615

[30] Alves M, Reinach PS, Paula JS, Vellasco ECAA, Bachette L, Faustino J, et al Comparison of diagnostic tests in distinct well-defined conditions related to dry eye disease. PloS one. 2014;9(5):e97921 10.1371/journal.pone.0097921 24848115 PMC4029783

[31] Astor FC, Hanft KL, Ciocon JO. Xerostomia: a prevalent condition in the elderly. Ear, nose, & throat journal. 1999;78(7):476–9. 10429321

[32] Saleh J, Figueiredo MA, Cherubini K, Salum FG. Salivary hypofunction: an update on aetiology, diagnosis and therapeutics. Arch Oral Biol. 2015;60(2):242–55. 10.1016/j.archoralbio.2014.10.004 25463902

[33] Villa A, Connell CL, Abati S. Diagnosis and management of xerostomia and hyposalivation. Therapeutics and clinical risk management. 2015;11:45–51. 10.2147/TCRM.S76282 25653532 PMC4278738

[34] Truong S, Cole N, Stapleton F, Golebiowski B. Sex hormones and the dry eye. Clinical & experimental optometry: journal of the Australian Optometrical Association. 2014;97(4):324–36. 10.1111/cxo.12147 24689906

[35] Wardrop RW, Hailes J, Burger H, Reade PC. Oral discomfort at menopause. Oral Surg Oral Med Oral Pathol. 1989;67(5):535–40. 2497421 10.1016/0030-4220(89)90269-7

[36] Syrjanen S. Age-related changes in structure of labial minor salivary glands. Age Ageing. 1984;13(3):159–65. 6731173 10.1093/ageing/13.3.159

[37] De Wilde PC, Baak JP, van Houwelingen JC, Kater L, Slootweg PJ. Morphometric study of histological changes in sublabial salivary glands due to aging process. Journal of clinical pathology. 1986;39(4):406–17. 3700674 10.1136/jcp.39.4.406 PMC499837

[38] Nagler RM. Salivary glands and the aging process: mechanistic aspects, health-status and medicinal-efficacy monitoring. Biogerontology. 2004;5(4):223–33. 15314272 10.1023/B:BGEN.0000038023.36727.50

[39] Vissink A, Spijkervet FK, Van Nieuw Amerongen A. Aging and saliva: a review of the literature. Special care in dentistry: official publication of the American Association of Hospital Dentists, the Academy of Dentistry for the Handicapped, and the American Society for Geriatric Dentistry. 1996;16(3):95–103. 10.1111/j.1754-4505.1996.tb00842.x 9084322

[40] Villa A, Nordio F, Gohel A. A risk prediction model for xerostomia: a retrospective cohort study. Gerodontology. 2015. 10.1111/ger.12214 26575829

[41] Villa A, Abati S. Risk factors and symptoms associated with xerostomia: a cross-sectional study. Australian dental journal. 2011;56(3):290–5. 10.1111/j.1834-7819.2011.01347.x 21884145

[42] Pajukoski H, Meurman JH, Halonen P, Sulkava R. Prevalence of subjective dry mouth and burning mouth in hospitalized elderly patients and outpatients in relation to saliva, medication, and systemic diseases. Oral surgery, oral medicine, oral pathology, oral radiology, and endodontics. 2001;92(6):641–9. 11740482 10.1067/moe.2001.118478

[43] Eidet JR, Chen X, Utheim TP, Utheim ØA, Stojanovic A, Stojanovic F, et al. Correlations between severity of dry eye symptoms and the use of systemic prescription drugs in a cohort of dry eye patients. The 7th International Conference on the Tear Film & Ocular Surface: Basic Science and Clinical Relevance Taormina, Sicily. 2013.

[44] Plemons JM, Al-Hashimi I, Marek CL, American Dental Association Council on Scientific A. Managing xerostomia and salivary gland hypofunction: Executive summary of a report from the American Dental Association Council on Scientific Affairs. Journal of the American Dental Association. 2014;145(8):867–73. 10.14219/jada.2014.44 25082939

[45] Schaumberg DA, Nichols JJ, Papas EB, Tong L, Uchino M, Nichols KK. The international workshop on meibomian gland dysfunction: report of the subcommittee on the epidemiology of, and associated risk factors for, MGD. Investigative ophthalmology & visual science. 2011;52(4):1994–2005. 21450917 10.1167/iovs.10-6997e PMC3072161

[46] Wise RJ, Sobel RK, Allen RC. Meibography: A review of techniques and technologies. Saudi J Ophthalmol. 2012;26(4):349–56. 10.1016/j.sjopt.2012.08.007 23961019 PMC3729652

[47] Matsumoto Y, Shigeno Y, Sato EA, Ibrahim OM, Saiki M, Negishi K, et al The evaluation of the treatment response in obstructive meibomian gland disease by in vivo laser confocal microscopy. Graefe's archive for clinical and experimental ophthalmology = Albrecht von Graefes Archiv fur klinische und experimentelle Ophthalmologie. 2009;247(6):821–9. 10.1007/s00417-008-1017-y 19101718

